# The repeatability of the abbreviated (4-h) Oral Fat Tolerance Test and influence of prior acute aerobic exercise

**DOI:** 10.1007/s00394-016-1320-z

**Published:** 2016-10-14

**Authors:** A. F. O’Doherty, T. Sathyapalan, A. S. Rigby, L. Ingle, S. Carroll

**Affiliations:** 1 Sport, Health and Exercise Science, University of Hull, Hull, UK; 2 Academic Diabetes, Endocrinology and Metabolism, Hull York Medical School, University of Hull, Hull, UK; 3 Centre for Cardiovascular and Metabolic Research, Hull York Medical School, University of Hull, Hull, UK

**Keywords:** OFTT, Postprandial metabolism, Acute exercise, Lipids, Repeatability

## Abstract

**Purpose:**

The Oral Fat Tolerance Test (OFTT) is regarded as a repeatable measure used to assess postprandial triglyceride (TAG) levels, with higher levels observed in cardio-metabolic disorders. Acute aerobic exercise intervention before OFTT reduces the TAG response, but the repeatability of this effect is unknown. The aim of this study was to determine the repeatability of the abbreviated 4-h OFTT with and without immediate prior aerobic exercise.

**Methods:**

On four separate days, healthy adult male participants underwent two 4-h OFTT (*n* = 10) and another two 4-h OFTT with 1-h of standardised moderate intensity aerobic exercise performed immediately before meal ingestion (*n* = 11). The OFTT meal composition included 75.4 g total fat, 21.7 g carbohydrate and 13.7 g protein. Venous blood was sampled at baseline and hourly up to 4 h after the OFTT meal ingestion, and TAG area under the curve (AUC) was calculated.

**Results:**

Nonparametric Bland–Altman analysis of 4-h TAG AUC revealed that 9 of 10 repeat measurements fell within ±15 % of the median TAG AUC for the OFTT. By contrast, two of 11 repeat measurements fell within ±15 % of the median TAG AUC for the OFTT undertaken with 1-h prior aerobic exercise.

**Conclusions:**

The 4-h OFTT is a repeatable test of postprandial TAG responses in healthy men. However, aerobic exercise performed immediately before OFTT considerably increases the variability of TAG AUC. These findings have implications for interpretation of research studies investigating exercise intervention performed immediately before OFTT. Future studies should also investigate the repeatability of exercise performed 8–24 h before OFTT.

## Introduction

The Oral Fat Tolerance Test (OFTT) is used to assess the capacity to adapt postprandial metabolic processes after a predefined oral fat load and evaluate cardio-metabolic health [1–5]. After oral fat consumption (>15 g), triglyceride (TAG) levels rise in the blood, typically peaking at 3–4 h and returning to baseline 6–8 h later [6]. These rises are exacerbated in those with cardiovascular and metabolic disorders and are associated with progression of atherosclerosis [1, 6, 7]. Since humans spend most of the day in the postprandial state, OFTT may reveal cardio-metabolic dysfunction not detected by traditional fasting measures [6, 8, 9].

At present, the OFTT is not widely used in a clinical setting to assess cardio-metabolic function. One reason for this could be the time demands of OFTT [10] which typically require postprandial measurements to be taken every 1 or 2 h for 5–8 h [11–16]. However, recently an abbreviated OFTT (lasting 4-h) has been developed and validated [3, 10]. This test would reduce the time constraints, improve the practicality of OFTT [10] and has been recommended by an expert consensus as clinically relevant and the most representative time to measure postprandial TAG responses following an OFTT [5]. Furthermore, the OFTT meal constitution (fat and carbohydrate content) is inconsistent across research studies that have used OFTT to induce postprandial lipaemia. Expert panel guidelines have also recommended OFTT meals to contain approximately 75 g fat, 25 g carbohydrate and 10 g protein [5]. Understanding the repeatability of OFTT is central to implementing this test in clinical and research environments. Postprandial TAG after OFTT is reported to have high repeatability in healthy [2, 10, 17] and overweight or obese adult participants [2, 10]. However, the statistical measures of agreement employed in these studies could be conceived as misleading with respect to attaining clinically meaningful measurement for repeatability, as discussed by Bland and Altman [18]. Therefore, an assessment of the repeatability of the 4-h OFTT with the meal composition meeting recommended guidelines and agreement assessed using Bland–Altman analyses is required.

Due to the relationship between elevated postprandial TAG and increased cardio-metabolic risk, interventions to acutely reduce postprandial lipaemia have been investigated. Acute aerobic exercise performed within 24 h of OFTT has often been shown to be an effective intervention in reducing postprandial TAG (for reviews see [19, 20]). An under-researched area, where conflicting data exist, relates to exercise performed shortly before OFTT to lower postprandial stresses [12–16, 21, 22]. Exercise performed shortly before OFTT either lowers [12, 15, 21] or has no effect on reducing postprandial lipaemia [13, 14, 16, 22]. To our knowledge, the repeatability of postprandial responses to OFTT with prior aerobic exercise (performed at any time point) has not been investigated. Due to the small sample sizes in the above-cited studies, if exercise has a highly variable within-person effect, this could account for the inconsistencies in the literature. Therefore, understanding the variability of postprandial TAG after OFTT with prior exercise is paramount for study design (sample size calculation) and interpretation of these data. An assessment of the repeatability of postprandial TAG to an abbreviated OFTT after acute exercise is required to address this issue.

The aim of this study was to investigate the repeatability of postprandial TAG after an abbreviated 4-h OFTT with and without prior aerobic exercise in apparently healthy adult males.

## Methods

### Participants

Apparently healthy adult males volunteered for this study. Participants were excluded if they had a past medical history of cardiovascular disease, gastrointestinal disease, liver disease, lipid lowering medication, hypertension, smoking, diabetes or family history of type 2 diabetes. This study was conducted according to the declaration of Helsinki and approved by the Department of Sport, Health and Exercise Science Ethics Committee, University of Hull. Written informed consent was given by all participants prior to commencing in the study.

### Study design

This prospective randomised crossover study investigated the repeatability of acute postprandial lipaemic responses (serum TAG concentrations) under two experimental conditions: (1) OFTT rest condition and (2) OFTT undertaken immediately after continuous aerobic exercise. Participants attended the research laboratory before 10:00 am on five separate occasions: one screening visit, two visits under the rest condition and two visits under the exercise condition. Each visit was separated by at least 72 h. The order in which the trial conditions were performed was randomised a priori for each participant using Research Randomizer software [23]. Participants refrained from alcohol and exercise for 24 h before each visit and attended the research laboratory having fasted overnight. All tests were completed within 8 weeks of the screening visit.

### Screening visit

Baseline height (Harpenden Stadiometer, Holtain Limited, Crymych Pembrokeshire), body mass (Seca 635 platform scales, Hamburg, Germany), waist and hip circumferences (Seca 201 ergonomic circumference measuring tape, Hamburg, Germany) in line with ACSM’s Guidelines for Exercise Testing and Prescription [24] and estimated body fat percentage using bioimpedance (BF900 Maltron Body Composition Analyser, Essex, UK) were recorded. Participants then underwent a 2-h oral glucose tolerance test (OGTT) with blood samples taken every 30 min for 120 min. Finally, participants performed a cardiopulmonary exercise test (CPET), as detailed below.

### Visits 1–4

An evening meal (as outlined below) was provided by the research team and consumed by the participant at home (unsupervised) on the evening before each laboratory visit. Participants fasted overnight (>12 h) and attended the laboratory the following morning. Baseline measures taken on the screening visit (detailed above) were repeated. Participants performed standardised continuous moderate intensity aerobic exercise, if randomised to the exercise condition, and consumed an OFTT meal immediately afterwards. Blood samples were taken at 1-, 2-, 3- and 4-h time points after the OFTT meal was consumed. An abbreviated 4-h time period to assess the postprandial response to OFTT was selected following the initial work of Weiss and colleagues [10] which has since been validated by Maraki and colleagues [3].

### Oral glucose tolerance test

Seventy-five grams of dextrose diluted in 300 ml of water was orally ingested by the participant, and blood samples were drawn at baseline and at 30-min intervals for 120 min after ingestion.

### Cardiopulmonary exercise test

Participants performed an incremental ramp-based CPET to volitional exhaustion on an electronically braked cycle ergometer (eBike ergometer, GE Healthcare, Freiburg, Germany) according to standard guidelines [25]. Online breath-by-breath expired gas analysis (Cortex Metalyzer 3B, Leipzig Germany) and 12 lead ECG (GE CASE system, GE Healthcare, Freiburg, Germany) was recorded throughout the test.

Peak oxygen consumption (${\dot{\text{V}}\text{O}}_{2}$ peak) was determined by identifying the highest period of oxygen consumption achieved by each participant averaged over 30 s [26]. The ventilatory anaerobic threshold (AT) was determined using the modified V-slope method [27] and confirmed with the ventilatory equivalents method [28].

### Continuous moderate intensity aerobic exercise

On two of the four study visits, a 1-h continuous moderate intensity aerobic exercise protocol was performed immediately before OFTT meal ingestion. After 3 min of rest, participants cycled at 20 watts (W) for 6 min, after which work rate was quickly increased to elicit 90 % of oxygen consumption at anaerobic threshold (90 % AT) which was maintained for 45 min. A 6 min “cool down” at 20 W completed the exercise protocol. A self-selected cadence during CPET was encouraged during steady state exercise.

A work rate in the moderate intensity domain [29] eliciting 90 %AT was calculated by identifying the work rate at AT from CPET, subtracting 2/3 of the ramp rate (to account for discrepancy between external work, muscle energetics and expired oxygen measured at the mouth [30]) and calculating 90 % of the value. Expired gasses were sampled throughout the exercise intervention; oxygen consumption and carbon dioxide measurements were used to estimate energy expenditure using the equations proposed by Jeukendrup and Wallis [31].

### Evening meal

To reduce the effects of meal composition on postprandial response to OFTT [32], participants chose one meal from a selection of evening meals. The mean (SD) nutritional contents of the meals were: calories 738 ± 18 kcal, protein 36 ± 1 g, carbohydrates 70 ± 9 g, fat 33 ± 2 g and saturated fat 12 ± 3 g. The same meal was provided to the participant for consumption on the evening before each study visit.

### Oral Fat Tolerance Test

The non-proprietary meal (Table 1) was designed specifically for this investigation and made primarily with dairy products and flavoured with chocolate powder. The high-fat meal was designed for participant palatability and in accordance with OFTT expert statement guidelines which recommended 75 g fat, 25 g carbohydrates and 10 g protein [5].

**Table 1:** Oral Fat Tolerance Test meal composition

Volume (ml)	375.0
Energy (kcal)	822.8
Fat (g)	75.4
Saturated fat (g)	47.4
Monounsaturated fat (g)	19.1
Polyunsaturated fat (g)	2.7
Trans fat (g)	2.4
Carbohydrate (g)	21.7
Protein (g)	13.7
Fibre (g)	2.0

### Blood sampling and analysis

Blood samples were drawn from a 20 gauge peripheral venous cannula (Braun Introcan Safety 20G Closed Catheter, Pennsylvania, USA) inserted into a vein in the lower arm. The cannula was kept patent between blood draws with a mandarin stylet (Braun Vasofix Stylet, Pennsylvania, USA). Up to 25 ml of blood was drawn at each time point. SST II blood collection tubes were stored at room temperature to clot for 30 min and then spun at 1992 g for 10 min at 4 °C.

The ABX Pentra 400 biochemistry autoanalyser (Horiba, Montpellier, France) was used to analyse serum triglycerides, total cholesterol, high-density lipoprotein cholesterol (HDL-C), apolipoproteins A1 and B, and glucose. Calibration and quality controls were performed in accordance with manufacturer’s guidelines prior to use, and samples were measured in duplicate. Low-density lipoprotein (LDL-C) was estimated from the Friedewald equation [33]. Serum insulin was measured using an ultrasensitive insulin assay on a Beckman Coulter DXI analyser (Beckman Coulter, inc, California, USA). The analyser is assessed as part of a UK national external quality assessment scheme (EQAS), total imprecision was <12 % across all analytical ranges.

### Outcome measures

The primary outcome for this study was postprandial TAG AUC following the 4-h OFTT. Secondary outcome measures were AUC for apolipoprotein B, glucose and insulin after 4-h OFTT.

### Statistical analysis

Normal (Gaussian) distribution of data was verified using the Shapiro–Wilk test, tests for skewness and kurtosis of distributions and visual inspection of histogram charts. Non-normally distributed data were transformed and analysed using parametric statistics where possible, and nonparametric analyses were performed when after transformation criteria for normal distribution, stated above, was not met. Data are presented as mean and standard deviation (SD) for normally distributed data, and non-normally distributed data are presented as median and quartiles 1 and 3 (Q1, Q3). AUC was determined by the trapezoidal method [34]. To determine agreement between repeated measures, Bland–Altman plots were used [18]; 95 % limits of agreement were estimated for parametric analyses. When data were non-normally distributed and could not be transformed, we offer both the parametric and nonparametric approaches to the Bland–Altman plot. Bland and Altman state, “if there are one or more extreme discrepancies between the methods, a nonparametric approach may be felt preferable” [18]. As such, nonparametric Bland–Altman methods with predefined arbitrary limits of agreement set to assess how many data points fell within these arbitrary limits were used. This provides a simple and more appropriate method for the reader to interpret repeatability in non-normally distributed data. An arbitrary limit of 15 % of the median TAG AUC was selected because exercise interventions typically reduce postprandial TAG by ≥15 % [19], therefore setting the upper limit of acceptable repeatability for exercise intervention studies. An arbitrary 10 % limit was set in accordance with the findings of Gill and colleagues who reported a 10 % variation for within-person postprandial TAG responses to OFTT in men [17]. We have also used Spearman’s ranked correlations and a novel statistical method proposed by Ryan and colleagues [2], which does not assume normality, to assess variability of TAG response to OFTT. Microsoft Excel (2013) and SPSS (Version 22) (SPSS Inc., Chicago, IL, USA) were used for all statistical analyses. Whole-body insulin sensitivity was estimated from relevant insulin and glucose measurements during the oral glucose tolerance test using the Matsuda index [35].

A sample size of 11 male participants was proposed by Gill and colleagues to detect a 10 % change (*α* = 0.05 and 80 % power) in TAG in intervention studies incorporating OFTT [17]. We selected this sample size to investigate the within-person variation prior to incorporating it into our future prospective interventional studies targeting a reduction in TAG AUC after OFTT.

## Results

Eleven apparently healthy males, median (Q1, Q3) age 30 (27, 44) years, mean (SD) body mass 79.0 (14.6) kg, and body mass index (BMI) 24.6 (3.5) kg m^−2^, were assessed, and participant demographics are reported in Table 2. Data for one participant were excluded for the rest condition due to breach of inclusion criteria, the participant stated (at the end of the study visit) that they had consumed alcohol within 24 h of the test. Accordingly, ten complete datasets are reported for the rest condition. Intra-individual variation of baseline fasting triglyceride concentration derived from the four fasting measurements (*n* = 10) was 19.1 %.

**Table 2:** Baseline demographics [mean (SD)]

Number of participants	11 male
Age (years)^a^	30.0 (27, 44)
Weight (kg)	78.3 (9.7)
BMI (kg m^−2^)	25.3 (3.1)
Waist hip circumferences ratio	0.86 (0.06)
Body fat content (%)^a^	21.0 (5.0)
${\dot{\text{V}}\text{O}}_{2}$ peak (ml kg^−1^ min^−1^)^a^	32.5 (32.0, 37.4)
AT (ml kg^−1^ min^−1^)^a^	15.7 (14.8, 18.5)
Matsuda index	10.1 (4.7)
HOMA IR	0.97 (0.41)
Triglyceride (mmol l^−1^)^a^	1.0 (0.7, 1.3)
Cholesterol (mmol l^−1^)^a^	5.4 (4.5, 5.6)
HDL-C (mmol l^−1^)^a^	1.4 (1.3, 1.5)
LDL-C (mmol l^−1^)^a^	3.4 (2.9, 3.8)
Apo B:Apo A1	0.73 (0.08)

^a^Median (Q1, Q3)

### Serum triglyceride response to OFTT

**Fig. 1:**
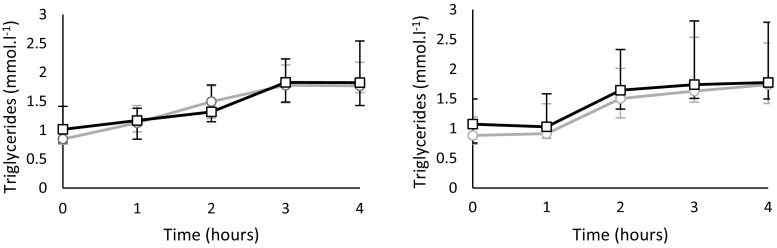
Median (Q1, Q3) triglyceride responses to OFTT during each trial. Left: *Circles* (*light grey line*) denote the first OFTT and *squares* (*black line*) the second OFTT under the rest condition (*n* = 10). Right: *Circles* (*light grey line*) denote the first OFTT and *squares* (*black line*) denote the second OFTT under the exercise condition (*n* = 11)

**Fig. 2:**
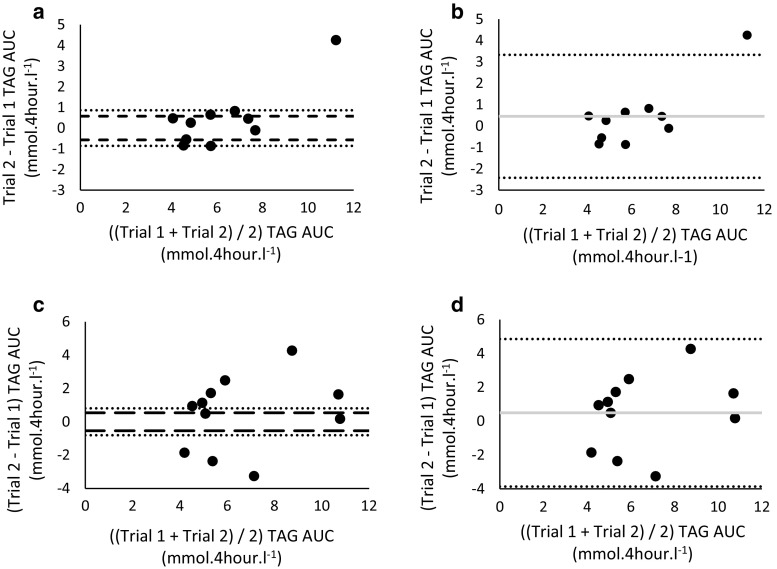
Alternative Bland–Altman analyses of postprandial triglyceride responses following rest and exercise conditions. Nonparametric Bland–Altman plots of postprandial triglyceride AUC after rest condition (*panel* **a**, *n* = 10) and exercise condition (*panel* **c**, *n* = 11). *Dashed lines* denote ±10 %, and *dotted lines* denote ±15 % of the median triglyceride area under curve. Parametric Bland–Altman plots of triglyceride AUC after rest condition (*panel* **b**, *n* = 10) and exercise condition (*panel* **d**, *n* = 11). *Dotted lines* denote 95 % limits of agreement (LOA) and *grey lines* denote the mean bias for each condition

**Fig. 3:**
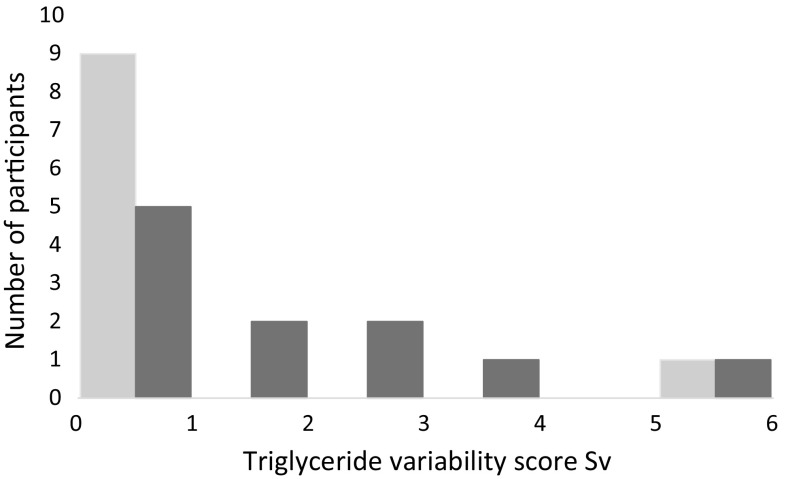
Variation score for individual participants for the rest condition (*light grey*, *n* = 10) and the exercise condition (*dark grey*, *n* = 11)

Figure 1 shows the median and quartiles 1 and 3 for TAG responses at each time point during OFTT. Median TAG showed incremental increases during the resting and postexercise OFTT attaining a peak concentration at 3–4 h. Figure 2 shows nonparametric Bland–Altman plots for TAG AUC for the repeated tests under each trial condition: rest (Fig. 2a) and exercise condition (Fig. 2c). To assess repeatability, limits of agreement were predefined at ±10 and ±15 % of the median score of all tests. For the rest condition, the limits of agreement for ±10 % of the median were −0.57 to 0.57 mmol 4 h^−1^l^−1^ and for ±15 % of the median were −0.86 to 0.86 mmol 4 h^−1^l^−1^. For the exercise condition, the limits of agreement for ±10 % of the median were −0.54 to 0.54 mmol 4 h^−1^l^−1^ and for ±15 % of the median were −0.81 to 0.81 mmol 4 h^−1^l^−1^. Five of ten data points fell within the 10 % limits of agreement for the rest condition and two of the eleven data points for the exercise condition. Nine of ten data points fell within the 15 % limits of agreement for the rest condition and two of eleven data points for the exercise condition. Parametric Bland–Altman plots for the rest and exercise condition are shown in Fig. 2b and d, respectively. The mean bias for the rest condition was 0.46 (95 % CI −0.59 to 1.51), and 95 % LOA were −2.42 (95 % CI −3.89 to −0.95) to 3.33 (95 % CI 1.87–4.80) mmol 4 h^−1^ l^−1^. The mean bias for the exercise condition was 0.49 (95 % CI −1.01 to 1.99), and 95 % LOA were −3.87 (95 % CI −6.89 to −0.85) to 4.85 (95 % CI 1.83–7.87) mmol 4 h^−1^ l^−1^. We assessed the Spearman’s ranked correlation between AUC of trial 1 and trial 2 of the rest and exercise conditions (Table 3a, b). Spearman’s ranked correlations were *ρ* = 0.90 and *ρ* = 0.42 for the rest and exercise conditions, respectively. Five participants had the same rank on each test (rank difference = 0) in the rest trial leading to a high ranked correlation coefficient of 0.90, whereas none of the participants had the same rank for the exercise condition. A variation score was calculated to assess repeatability, and the variation of this score across the groups can be observed in Fig. 3. The closer the score is to 0, the smaller the variation of the measure. Nine of the 10 data points scored <1 for the rest condition compared to 5 of 11 data points for the exercise condition.

**Table 3:** Spearman’s ranked correlation for triglyceride AUC in the (a) rest condition, (b) exercise condition

	Rest 1	Rest 2	Rank 1	Rank 2	Difference	Difference^2^
*(a) Rest condition*						
R09	3.83	4.30	1	2	−1	1
R06	4.72	4.98	2	4	−2	4
R08	4.92	4.37	3	3	0	0
R11	4.95	4.11	4	1	3	9
R05	5.39	6.04	5	6	−1	1
R10	6.16	5.30	6	5	1	1
R03	6.37	7.19	7	7	0	0
R07	7.14	7.60	8	8	0	0
R02	7.73	7.63	9	9	0	0
R01	9.09	13.35	10	10	0	0
				Sum		16
				Sum × 6		96
				(Sum × 6)/*n*(*n* ^2^−1)		0.10
				Rank correlation		0.90
*(b) Exercise condition*						
R11	4.05	5.00	1	3	−2	4
R06	4.38	5.51	2	6	−4	16
R05	4.45	6.17	3	7	−4	16
R03	4.67	7.15	4	8	−4	16
R04	4.83	5.32	5	4	1	1
R08	5.12	3.27	6	1	5	25
R09	6.56	4.20	7	2	5	25
R10	6.61	10.88	8	10	−2	4
R02	8.76	5.51	9	5	4	16
R01	9.88	11.52	10	11	−1	1
R07	10.68	10.86	11	9	2	4
				Sum		128
				Sum × 6		768
				(Sum × 6)/*n*(*n* ^2^ − 1)		0.58
				Rank correlation		0.42

### Glucose, insulin and apolipoprotein B responses to OFTT

**Fig. 4:**
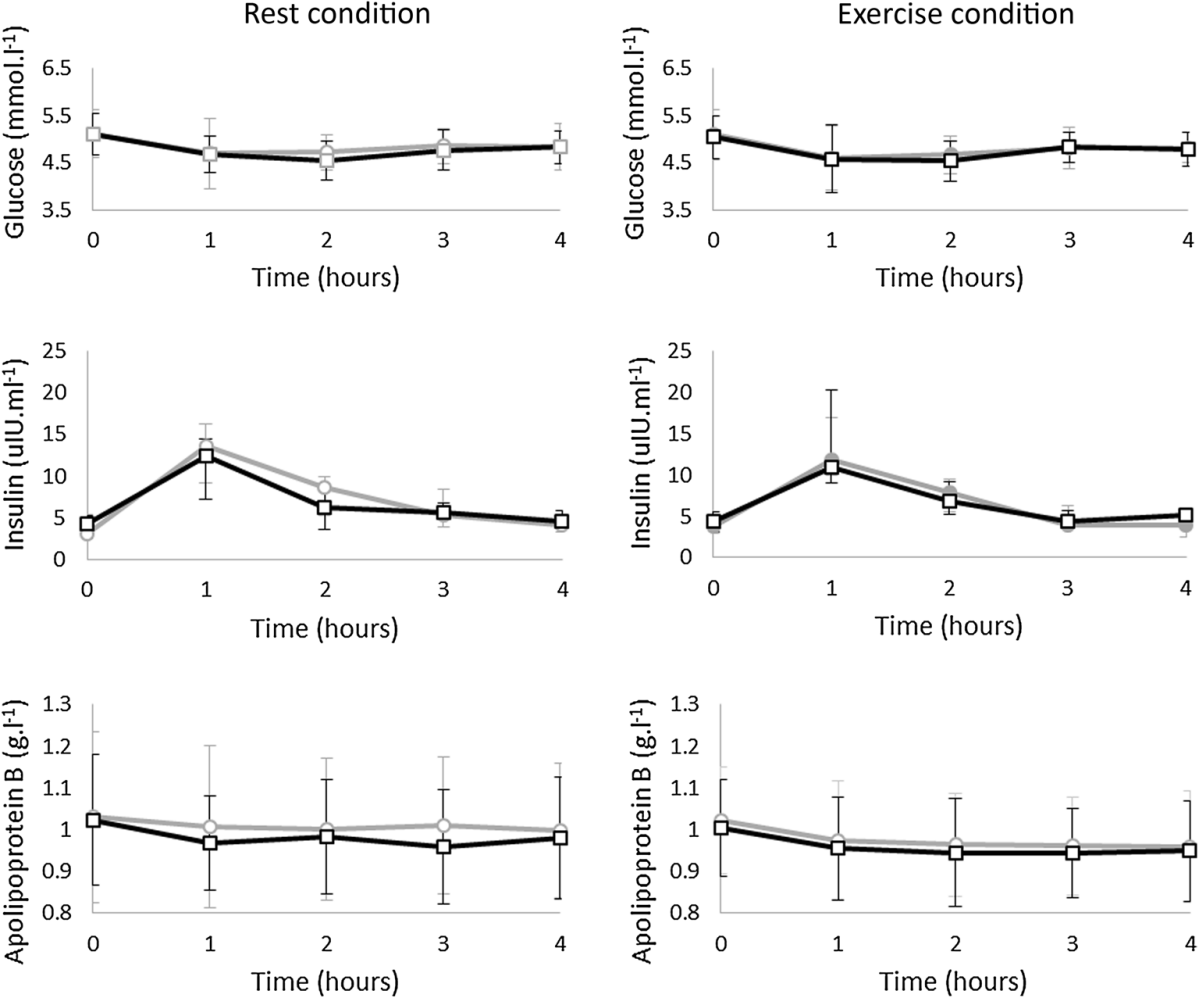
Mean (SD) glucose responses (*top panels*), median (Q1, Q3) insulin responses (*middle panels*) and mean (SD) apolipoprotein B responses (*bottom panels*) to OFTT during each trial. Left panels: *Circles* (*light grey line*) denote the first OFTT and *squares* (*black line*) the second OFTT under the rest condition (glucose and apolipoprotein, *n* = 10; insulin, *n* = 9). Right panels: *Circles* (*light grey line*) denote the first OFTT and *squares* (*black line*) denote the second OFTT under the exercise condition (*n* = 11)

Figure 4 shows the 4-h responses of glucose, insulin and apolipoprotein B to the OFTT. The limits of agreement (LOA) and mean bias for apolipoprotein B, glucose and insulin are reported in Table 4. Insulin AUC was not reported for one participant in the rest condition due to haemolysis of a serum sample at the 1-h time point; insulin AUC data are reported on 9 participants.

**Table 4:** Repeatability of glucose, insulin and apolipoprotein B 4 h AUC after Oral Fat Tolerance Test

Substrate	Grand mean	SD	Mean bias	Lower 95 % LOA	Upper 95 % LOA
*Glucose (mmol* *4* *h* ^−*1*^ *l* ^−*1*^ *)*					
Rest condition	18.88	1.13	−0.26	−2.40	1.89
Exercise condition	19.10	1.35	−0.07	−2.50	2.36
*Insulin* (*uIU* *4* *h* ^−*1*^ *ml* ^−*1*^ *)* ^a^					
Rest condition^b^	33.34	16.47	0.91	0.70	1.18
Exercise condition	31.02	12.10	1.03	0.62	1.73
*Apo B (g* *4* *h* ^−*1*^ *l* ^−*1*^ *)*					
Rest condition	3.97	0.59	−0.12	−0.85	0.60
Exercise condition	3.86	0.47	−0.07	−0.46	0.33

95 % LOA are the 95 % limits of agreement, *SD* standard deviation

^a^Insulin data were log transformed; therefore, geometric mean and SD are reported, and mean bias with 95 % LOA is reported as a ratio

^b^Insulin AUC for OFTT *n* = 9

### Continuous moderate intensity aerobic exercise

All participants completed the continuous moderate intensity aerobic exercise intervention on a cycle ergometer, on two separate days, immediately before high-fat meal ingestion. Median (Q1, Q3) work rate at 90 % AT was 70 (67, 76) W. Median (Q1, Q3) estimated energy expenditure was 250 (221, 252) kcal on the first exercise intervention and 243 (230, 269) kcal on the second exercise intervention. Median respiratory quotient during exercise at 90 %AT was 0.90 (0.88, 0.92) and 0.89 (0.87, 0.91) for the first and second exercise intervention, respectively. Median heart rate was 105 (102, 109) and 104 (102, 110) beats min^−1^ for the first and second exercise intervention, respectively. Accordingly, the metabolic responses were consistent between the first and second acute exercise interventions.

## Discussion

The results from the present study demonstrate that 4-h TAG responses to OFTT in men are repeatable; however, when aerobic exercise is performed immediately before OFTT the repeatability is poor. Secondary findings demonstrate that repeatability of glucose was good, with wider limits of agreement for both insulin and apolipoprotein B responses to OFTT (limits of agreement within ±11, −21 to 27 and ±18 % of the mean, respectively). Limits of agreement for glucose and apolipoprotein B after OFTT with prior exercise were good (within ±13 and ±10 % of the mean), but insulin showed very poor repeatability (LOA from −41 to +70 %).

We investigated the within-person variation of a previously validated abbreviated 4-h OFTT [3, 10]. The repeatability of TAG responses to OFTT with measurements taken for 5–8 h post-ingestion has been demonstrated previously; however, absolute measures of limits of agreement have not been reported [2, 10, 17, 36]. We identified with nonparametric Bland–Altman analyses that 90 % of the data points fell within ±0.86 mmol l^−1^ equating to ±15 % of the median. This value could be considered as a clinically meaningful change for intervention studies utilising 4-h OFTT. Spearman’s ranked correlations also highlight the strong relationship between the two rest trials (*ρ* = 0.90) and weaker relationship between the two exercise trials (*ρ* = 0.42). We tried to reduce variation by controlling the composition of the evening meal prior to the OFTT as this has been reported to alter the TAG response [32]. We also employed a recently proposed [2] assessment of variability in TAG responses to OFTT and identified that 9 of 10 participants had very low variation and one participant having very large variation between the two OFTTs. In keeping with the literature, higher fasting and peak TAG responses are prone to greater variation [2]. Such variation is not uncommon, Ryan and colleagues [2] reported variation in 18 % of their population and associate this variation or “deviation from the norm” [2] with phenotypic and genotypic characteristics. The proposed existence of a variable phenotype supports the statistical methods that we have employed to assess repeatability as these “outliers” cause a non-normal distribution which would over-estimate limits of agreement using parametric or log transformed Bland–Altman plots particularly with our small sample size.

Exercise interventions prior to OFTT have been shown to be effective in reducing postprandial TAG responses. However, to our knowledge, the repeatability of this effect has not been investigated prior to this study. We have shown that exercise performed immediately prior to high-fat meal ingestion provokes highly variable TAG responses after OFTT. This variation could explain the inconsistency in the findings of exercise intervention studies where immediate prior exercise has been shown to reduce [12, 15, 21] or have no effect [13, 14, 16, 22] on the postprandial TAG response. Caution should therefore be taken with regard to interpretation of studies employing aerobic exercise interventions immediately prior to OFTT. Implications of this finding for clinical practice would be limiting moderate or vigorous physical activity, such as a long walk to the hospital or clinic, immediately prior to OFTT.

We also assessed the agreement of these data using parametric Bland–Altman analyses and demonstrated poor agreement with both rest and exercise trial conditions (Fig. 2b, d). The assumptions of the parametric Bland–Altman approach are that data are normally distributed, that is 95 % of data falls within two Standard Deviations. Clearly, when data are non-normally distributed, as with our dataset, two standard deviations will not reflect 95 % of these data and therefore the limits of agreement calculated from two standard deviations have no relevance to these data. This is particularly apparent in Fig. 2b, where the apparent “outlier” markedly widens and therefore over-exaggerates the limits of agreement. The failure of our dataset to meet the fundamental assumptions of parametric analysis supports the use of the nonparametric methods selected and used to formulate our conclusions.

The OFTT meal composition for this study had a lower carbohydrate content than some exercise intervention studies [13, 14, 21, 22, 37] but similar to others [12, 15, 16]. Carbohydrate and protein enable lipid absorption and the quantities that we used were similar to the OFTT proposed in an expert panel statement that had considered lipid absorption in the design of the meal [5]. Carbohydrate ingestion stimulates increased insulin secretion which suppresses fatty acid oxidation in the liver and upregulates triglyceride removal from plasma into adipose and muscle tissue [38]. Increased insulin as a result of a high-fat (80 g) high-carbohydrate (100 g) meal evoked a lower postprandial TAG AUC compared with a high-fat (80 g) low-carbohydrate meal (20 g) in healthy participants [39]. Acute exercise is associated with improved whole-body insulin sensitivity for up to 48 h [40]. Therefore, the carbohydrate content of OFTT meals is an important consideration for acute exercise intervention studies incorporating OFTT, but to our knowledge this has not been investigated.

The variation in baseline TAG across the four testing days was consistent with literature on biological variation of TAG [41]. The limits of agreement for blood glucose suggested good repeatability as has been previously reported in OFTT [17]. The relatively low carbohydrate content of the meal led to small changes in glucose concentrations from baseline to 4 h. The repeatability of insulin AUC was poor. Our measurements indicated that insulin concentrations were highest at the 1-h time point during OFTT and returning to baseline concentrations at the 2-, 3- and 4-h time points. The apparent poor agreement in insulin AUC is most likely due to insufficient measurements taken around the peak circulating insulin time point, rather than the variability of the insulin response. Therefore, the hourly blood sampling time points of this study may not be appropriate to assess the postprandial insulin responses to OFTT. Apolipoprotein B showed good limits of agreement for the exercise condition with wider limits of agreement for the rest condition. Total apolipoprotein B responses to OFTT appear to be less susceptible to acute changes compared to triglyceride responses, consistent with previous findings among healthy, obese and hyperlipidaemic participants [42]. The within-person variability of apolipoprotein B to OFTT should be considered meaningful for future studies assessing this measure.

The strengths of this study include the robust study design which allowed investigation into the repeatability of OFTT under two conditions. The intensity of exercise was rigorously controlled, and previous OFTT exercise intervention studies have selected exercise intensities as a percentage of ${\dot{\text{V}}\text{O}}_{2}$ peak which does not necessarily control for exercise intensity domains. Furthermore, we attempted to control food ingestion on the evening before the OFTT visits. Limitations include the small sample size enrolled in the study and that we investigated a liquid meal rather than solid meal. To reduce the confounding effect of exercise other than that prescribed in the protocol, we asked participants not to exercise for 24 h before OFTT. This was in accordance with the findings of Zhang et al. [11] where only exercise conducted 12 h before OFTT and not 24 h before OFTT reduced postprandial TAG. However, other protocols required participants to refrain from exercise for up to 3 days before OFTT to remove the effect of prior exercise [12, 17, 22]. Therefore, we cannot rule out a confounding effect of exercise performed between 24 and 72 h before each OFTT.

In conclusion, our data indicate that the postprandial TAG response to the abbreviated 4-h OFTT in men is repeatable with relevant and clinically meaningful statistical evaluation of variability. However, acute aerobic exercise performed immediately prior to OFTT provokes highly variable within-person responses. Interpretation of data from studies investigating the effects of immediate prior acute exercise should be undertaken with caution. Future studies should investigate the repeatability of TAG responses to OFTT with exercise interventions performed 8–24 h before meal ingestion.
